# Nonspecific Interstitial pneumonia (NSIP)/ Overlap or Distinct Entity: A case report from the National Research Institute of Tuberculosis and Lung Disease (NRITLD)

**Published:** 2014

**Authors:** Payam Mehrian, Ali Cheraghvandi, Atousa Droudnia, Firouzeh Talischi, Saeid Fallah Tafti, Shahram Kahkouee, Hamidreza Jamaati

**Affiliations:** 1 Pediatric Respiratory Diseases Research Center, National Research Institute of Tuberculosis and Lung Diseases (NRITLD), Shahid Beheshti University of Medical Sciences, Tehran, Iran; 2 Lung Transplantation Research Center, National Research Institute of Tuberculosis and Lung Diseases (NRITLD), Shahid Beheshti University of Medical Sciences, Tehran, Iran; 3 Tracheal Diseases Research Center, National Research Institute of Tuberculosis and Lung Diseases (NRITLD), Shahid Beheshti University of Medical Sciences, Tehran, Iran.; 4 Nursing and Respiratory Health Management Research Center, National Research Institute of Tuberculosis and Lung Diseases (NRITLD), Shahid Beheshti University of Medical Sciences, Tehran, Iran.; 5 Tobacco Prevention and Control Research Center, National Research Institute of Tuberculosis and Lung Diseases (NRITLD), Shahid Beheshti University of Medical Sciences,Tehran, Iran.; 6 Mycobacteriology Research Center, National Research Institute of Tuberculosis and Lung Diseases (NRITLD), Shahid Beheshti University of Medical Sciences, Tehran, Iran.; 7 Chronic Respiratory Diseases Research Center, National Research Institute of Tuberculosis and Lung Diseases (NRITLD), Shahid Beheshti University of Medical Sciences, Tehran, Iran

**Keywords:** Lung Diseases, Interstitial Pneumonia; Diagnosis, Computed tomography

## Abstract

***Background: ***In many cases of ILD (interstitial lung disease), overlap diagnosis is considered. Here, a few cases with diagnosis of a variety of ILDs, where eventual open lung biopsy has been performed are selected. Reference will be made to reliable sources to show that NSIP can still be a variant of UIP (Usual interstitial pneumonia) with better treatment response and prognosis.

***Case Presentation:*** In case 1, there is a difference between the HRCT(High Resolution Computed Tomography) result (NSIP pattern without fibrosis) and pathologic result (which includes fibrosing NSIP more closely related to UIP).Case 2 shows obvious discord between HRCT result (UIP pattern) and pathologic result (NSIP pattern). In case 3, there is again a discrepancy between HRCT report (very mild architectural distortion suggestive for ILD like NSIP) and pathology report (destructed lung tissue with interstitial fibrosis suggestive of HP (Hypersesitivity Pneumonitis) and not NSIP.

***Conclusion: ***In this paper, we demonstrate that although NSIP can be a distinct diagnosis in most cases, but in rare cases the distinction between the other kinds of ILD especially UIP and NSIP in spite of full workup including tissue assessment can be very difficult.

In medicine, the term overlap is very common, to the extent that there is even a syndrome with this name. Overlap is also frequently seen in pulmonary fibrosis, such that expert pathologists use diagnoses such as UIP/sarcoidosis or UIP/HP (hypersensitivity pneumonitis) when interpreting cases of pulmonary fibrosis. Even though NSIP has distinguished etiologic, radiologic, pathologic, and response to treatment characteristics from other diagnoses in lung fibrosis particularly IPF (idiopathic pulmonary fibrosis), there is still a well-known overlap between NSIP and UIP. NSIP may overlap with other pulmonary fibrotic conditions when it comes to the clinical, radiologic and pathologic picture. In this article, we present a few cases and will make reference to reliable sources to show that NSIP is still a variant of UIP which has better treatment response and prognosis and there should be no insistence for using the term NSIP. The purpose of this paper was to discuss the difficulties in confirming clinical diagnosis of NSIP. Not only, the specific diagnostic radiologic signs on HRCT (high resolution computed tomography) such as in the case of UIP are missing (1-8), but we also face overlap diagnoses pathologically. Furthermore, there are cases where we even have specific pathologic diagnosis of NSIP, but it is hard to clinically accept it. 

According to the last ATS (American thoracic society) / ERS (European respiratory society) consensus panel (9) regarding idiopathic interstitial pneumonias, the more common causes of diffuse parenchymal lung disease (DPLD) can be easily distinguished clinically, radiologically and pathologically from IPF, granulomatous conditions such as sarcoidosis and other less common causes of DPLD such as LAM (lymphangiolyomyomatosis) and histiocytosis X. Further diagnostic categorization has led to terms such as NSIP and LIP (lymphocytic interstitial pneumonia). Only on a pathologic basis and also more recently radiologically, one can conclude that NSIP should not be placed as a distinct entity alongside with UIP. 

## Case Presentation

**Case One: **The patient is a 50-year old female with a history of hypothyroidism who presents with complaints of progressive dyspnea and arthralgia for the past 9 months. She denies any fever, chills, cough and sputum. She has been treated as an outpatient with levothyroxine, prednisolone, calcium and acetylcystein which has improved her dyspnea. Heart exam is normal. Lung exam shows fine crackles bilaterally at lung bases. She has 1+ edema of both lower extremities.

HRCT showed subpleural interlobular septal thickening with bilateral ground glass opacities predominantly in the lower zones suggestive of NSIP without fibrosis. The patient underwent TBLB (transbronchial lung biopsy) which showed interstitial pneumonia with eosinophilic infiltration. CBC was normal except for WBC of 11.7*10^3cells/ MicroL. ESR was 78 mm/h. Sputum smear was negative for AFB (acid fast bacillus), but positive for strep Viridans. Echocardiogram showed EF (ejection fraction) of 65% with no pulmonary hypertension. She subsequently underwent video assisted thoracoscopic lung biopsy. Pathology of RLL (right lower lobe) biopsy showed patchy random distortion of alveolar architecture. In involved areas, interstitium was thick due to prominent chronic lymphocytic inflammatory cells infiltration and fibrosis associated with some intraalvolar granulation tissue formation and diffuse pneumocyte type II hyperplasia. Pathology conclusion was consistent with NSIP pattern mixed cellular and fibrosing. The patient is discharged in good condition to be followed-up in the Outpatient clinics on medication with prednisolone 50 mg (1/3 tablet) with biopsy results (see above).

**Case Two: **The patient is a 41 year old male diabetic stone carver who has been nonsmoker for the last 20 years. He presents with progressive shortness of breath for the past 2 months. He was admitted with the diagnosis of UIP based on HRCT for completion of diagnostic work up. He noted periodic fevers and chills. Medications on admission were metformin, verapamil and propranolol. Heart and lung exams were normal. 

 Thorax CT showed bilateral predominantly lower lobe and peripheral lung opacities with interstitial septal thickening highly suggestive for UIP pattern. CBC was within normal limits. ESR was 42 mm/hr. RF and ANA were negative. Echocardiogram showed a PAP (pulmonary arterial pressure) of 35-40mmHg.

The patient underwent open lung biopsy. Pathology of lingula and LLL (left lower lobe) biopsy showed mildly distorted alveolar architecture due to diffuse interstitial chronic inflammatory cell infiltration associated with mild fibrosis and scattered Masson bodies, foci of follicular lymphoid aggregates, DIP (desquamative interstitial pneumonia) reaction, peribronchiolar foamy macrophage aggregation, prominent pneumocyte type II hyperplasia and a few subpleural honeycomb changes. There was no evidence of unquestionable fibroblastic foci or heterogeneous involvement of the lung tissue. Trichrome staining revealed no prominent collagenized fibrosis in interstitium. Conclusion of pathology report was NSIP pattern. Clinical, radiologic, and pathologic correlation were recommended. He was discharged in good condition. He has been subsequently followed-up in the Outpatient clinic and has been stable without symptoms on medication with Azathioprine and prednisolone.

**Case Three: **The patient is a 53-year old male who has been diagnosed and treated for COPD (chronic obstructive pulmonary disease) in the past 5 years. He presented with shortness of breath, cough and sputum. He has no weight loss. Three months prior to admission, he was hospitalized in his hometown where he received antibiotic therapy. He has history of smoking and oral opium intake. Medications at admission were salmeterol and atrovent. Cardiac exam was normal. Lung exam showed fine rales in the bases of the lungs and generalized wheezing. 

HRCT showed patchy irregular bilateral pulmonary opacities with very mild honey combing and architectural distortion and interlobular septal thickening suggestive for ILD such as induced by NSIP. CBC was within normal limits. ESR was 9mm/hr. Sputum for AFB was negative. Echo showed no pulmonary hypertention with EF of 55%. The patient was admitted with possible diagnosis of ILD or sarcoidosis. He underwent bronchoscopy. BAL (bronchoalveolar lavage) washing was negative for malignancy. Transbronchial lung biopsy showed mild infiltration of lymphocytes and anthracosis and bronchial mucosa had mild chronic nonspecific inflammation. The patient underwent open lung biopsy and the results of RLL biopsy showed distorted lung tissue due to areas of interstitial fibrosis, extensive bronchiolarization, chronic interstitial lymphocyte inflammatory cells infiltration and even lymphoid aggregation. There was intraalveolar and intrabronchiolar polypoid granulation tissue formation. The conclusion was destructed lung tissue with interstitial fibrosis, chronic inflammation, bronchiolitis and bronchiolarization suggestive of HP. 

The patient received full course of cefazolin and azithromycin antibiotics. With pathology result consistent with HP, he was started on treatment with corticosteroids. Shortness of breath, cough and sputum improved and he was discharged with prednisolone, omeprazole, bronchodilators and Ca-D to be followed-up in Outpatient clinics.

## Discussion

The distinct term of NSIP was first used by pathologists (Katzenstein) and later separately by radiologists. On the other hand, historically it has been shown that UIP has a worse prognosis; yet, some forms of it pathologically and radiologically show more inflammation which is more consistent with NSIP. Logically, it seems that on a clinical basis the two should not be separated (10). The few cases presented show that even pathologists use overlap diagnosis with NSIP and other idiopathic interstitial pneumonias (10, 11). The first case is more of a typical presentation of NSIP. Additionally, even with NSIP when it comes to pathology, radiology and clinic, substantial diversity exists (1).

Here, we discuss the pathologic, radiologic and clinical characteristics of UIP and NSIP so that the similarity between these two terms is better shown. As it has been agreed upon, the use of UIP with better prognosis is better than NSIP clinically, since clinicians believe that NSIP in its more inflammatory form responds better to treatment and there is a chance of recovery. UIP/IPF- This condition is mainly seen above the age of 50 in the form of fibrosis in the lung bases with known characteristic HRCT findings. Pathologically, the presence of patchy geographic distribution and heterogeneity found in biopsies from different areas are clearly seen in UIP. Yet, without the visualization of areas with fibroblasts, differentiation from other causes of end stage pulmonary fibrosis is difficult. On the other hand, the presence of foci of unformed granulomas, severe lymphocytic inflammation and follicle formation and unclear picture of organizing pneumonia, has made use of UIP even on a pathological basis unfree of clarity (7, 8, 12). NSIP patients are frequently women in younger ages. This condition can present with other underlying disorders such as collagen vascular diseases or due to medications and lead to pulmonary fibrosis and at the same time chronic HP. Many times the etiology is idiopathic. In HRCT compared to UIP, there is often less peripheral distribution and ground glass opacities consistent with inflammation seen. Pathologically, there is the uniformity of the interstitium, increased cellularity or fibrosis, but less honey combing. Yet, often it is difficult to distinguish fibrosing NSIP form IPF/UIP (13-15).

The remainder of the discussion refers to our case presentations regarding radiology and pathology when it comes to overlap of diagnoses. It seems that over expanded to consider NSIP as a variant of IPF with good prognosis. On the other hand, the assumption that in certain situations NSIP has turned into UIP has not been shown with enough evidence. Yet, when NSIP is described similarly in terms of pathology and radiology, clinically it is probably not clear cut to distinguish it from UIP. Even researchers like Katzenstein et al. who first described NSIP believe that primary tissue injury that leads to UIP can at times lead to inflammation and fibrosis and produce the picture of NSIP (16). Their evidence is the presence of foci of pathologic findings similar to NSIP in UIP patients. Yet, we do not want to make the same error for years when DIP was considered a spectrum of IPF. We still believe that clinically, to use the term NSIP, stronger evidence is needed (17).

Is it appropriate clinically to use NSIP only in rheumatologic conditions with pulmonary findings? It should be noted that in rheumatologic conditions, only NSIP pattern is not found and sometimes LIP or UIP particularly in patients with Sjorgen or rheumatoid arthritis can be seen (18,19). On the other hand, the response to the question of whether NSIP in rheumatologic diseases is the same as idiopathic NSIP is very difficult, particularly since in pathologic samples the signs of bronchiectasis or granuloma may not be found (10, 14). Overall, the ATS/ERS workshop committee has used the logical diagnosis of NSIP for middle aged women and nonsmokers without clinical and serologic evidence for collagen vascular disease (2). Even in some situations, NSIP has clinically been used in cases of undifferentiated connective tissue disorder (20).

We believe that using the term NSIP and UIP provides the suggestion that in NSIP, steroids have absolute indication and the prognosis is better. On the other hand, UIP has poor prognosis and steroids are not useful. Yet, this is not the practical approach and the management of patients is individually-based. 

It has previously been supported that NSIP is used for the cases discussed above. In the ATS/ERS 2002 categorization, grey area exists in this differentiation. The purpose of using distinct diagnostic entities due to variability in pathology, clinics and prognostics has been accepted. It can be concluded, that NSIP has been pathologically accepted as a distinct entity, but clinically it is used for middle aged women, nonsmokers with clear picture pathologically and on HRCT pattern consistent with NSIP (7, 17, 21). In the diagnosis of NSIP, not only there is a need for a group approach between the clinician, radiologist and pathologist, but clinically the presence of underlying collagen vascular disease strongly confirms the diagnosis and makes it acceptable.
